# Advancing dementia care through implementation science: global and Australian perspectives

**DOI:** 10.1002/alz70858_096548

**Published:** 2025-12-24

**Authors:** Anita M Y Goh, Ellen Gaffy, Sanne Peters, Cathy Roth, Briony Dow

**Affiliations:** ^1^ National Ageing Research Institute, Parkville, VIC, Australia; ^2^ The University of Melbourne, Parkville, VIC, Australia; ^3^ PALZ ‐ Professionals with Alzheimer's and related diseases, Geelong, VIC, Australia; ^4^ National Ageing Research Institute, Melbourne, VIC, Australia; ^5^ University of Melbourne, Parkville, VIC, Australia

## Abstract

**Background:**

Research has yielded advancements in understanding dementia and developing promising interventions. However, translating research findings into routine practice and policy remains a significant challenge. Additionally, there are few studies of implementation; that is, examination of the processes, outcomes, and factors influencing implementation of interventions. Implementation science provides a critical framework for addressing this gap by investigating factors that influence the adoption and sustainment of evidence‐based dementia care interventions in real‐world settings.

**Method:**

This presentation provides an up‐to‐date overview of the evolving field of dementia implementation science, highlighting its role in improving care outcomes. The presenter outlines key theoretical frameworks and the core principles of implementation science, including identifying and addressing barriers to implementation, developing and testing implementation strategies, and evaluating the effectiveness of these strategies. This presentation uses as an example an applied research example from Australia – the Promoting Independence Through quality dementia Care in The Home (PITCH) study, aimed to improve sense of competency in dementia home care and outcomes for people with dementia via a co‐designed evidence‐based dementia training program.

**Result:**

A national cluster stepped‐wedge randomized controlled trial tested PITCH, alongside a health economic and process evaluation. Overall, the interactive, adult‐learning‐based, face‐to‐face PITCH program was effective in improving sense of competence in dementia care as well as knowledge and attitudes. For carers and people with dementia, no differences in health and quality of life outcomes were found. The process evaluation explored barriers and facilitators to implementation of PITCH, explored its acceptability, feasibility, suitability, and sustainability, and whether participation led to changes in practice. Themes from the process evaluation (from qualitative interviews and surveys) investigating the experiences and opinions of the intervention receivers and intervention deliverers included their motivations to participate, perceived benefits of PITCH, challenges of involvement, and suggestions for improvement, analyzed using the Theoretical Domains Framework. Results inform how the PITCH intervention could be modified to improve future wider implementation.

**Conclusion:**

By applying rigorous methodologies, the field of implementation science offers a promising avenue for maximizing the impact of dementia research and enhancing the quality of life for individuals living with dementia and their caregivers.

